# A comprehensive diagnostic approach in suspected neurosarcoidosis

**DOI:** 10.1038/s41598-023-33631-z

**Published:** 2023-04-21

**Authors:** Shala Ghaderi Berntsson, Andreas Elmgren, Olafur Gudjonsson, Anna Grabowska, Anne-Marie Landtblom, Maria-Francisca Moraes-Fontes

**Affiliations:** 1 Department of Medical Sciences, Neurology, Uppsala University, 751 85 Uppsala, Sweden; 2 Department of Medical Sciences, Neurosurgery, Uppsala University, Uppsala, Sweden; 3 Department of Neuroradiology, Uppsala University, Uppsala, Sweden; 4 Fundação Champalimaud, Lisbon, Portugal

**Keywords:** Neuroscience, Neurology

## Abstract

Neurosarcoidosis presents a diagnostic challenge in clinical settings, as it has no pathognomonic symptoms or signs and a wide range of differential diagnoses. The aim of this report is to present the pathological features of our group of patients, obtained through a systematic diagnostic approach. This retrospective cohort study enrolled all adult patients primarily diagnosed with neurosarcoidosis at the neurology department of a tertiary center in Sweden over a period of 30 years, from 1990 to 2021. We identified 90 patients, 54 with possible neurosarcoidosis and 36 with probable neurosarcoidosis. CNS biopsy revealed an alternative diagnosis for 24 patients, who were then excluded. The collected data from medical records included demographic and clinical characteristics, systemic and/or neurological isolated involvement, various laboratory tests, including cerebrospinal fluid (CSF), serum analysis, imaging studies (MRI, FDG-PET/CT, and HRCT), nerve conduction studies, electromyography, and pathology reports of central nervous system (CNS), and extra-neural tissue biopsies. Sixty-six patients were included in our cohort. The median age at onset of symptoms was 49 years, with a similar sex distribution. Cranial neuropathies (38%), motor deficit (32%), headache (16%), and pituitary dysfunction (12%) were the most common presenting features. CSF studies were abnormal in 77% of the patients, who showed lymphocytosis (57%), elevated protein (44%), oligoclonal bands (40%), elevated ACE (28%), and raised T lymphocyte CD4^+^/CD8^+^ ratios (13%). Strikingly, MRI showed that 17% of the patients presented with isolated pituitary gland lesions. FDG-PET/CT was performed in 22 patients (33%) and confirmed systemic sarcoidosis in 11. Despite our extensive workup, the final classification for our patients only allowed for a definite diagnosis in 14 patients; the remainder were classified as probable (32) or possible (20) neurosarcoidosis. Since 2007, the employment of a structured laboratory and imaging approach and the increasing number of CNS biopsies have facilitated and improved the process of correct attribution in patients with presumptive neurosarcoidosis, especially in patients with isolated neurological lesions. We highlight a higher frequency of pituitary lesions due to neurosarcoidosis than has been classically described. A detailed laboratory diagnostic workup is included.

## Introduction

Sarcoidosis, a systemic granulomatous disorder of unknown etiology, was initially described in 1877 by Hutchinson^1^. It is a global disease with an incidence that differs among ethnic groups and sexes. It ranges in annual age-adjusted incidence from 0.2/100,000 in Brazil, to 19/100,000 in Sweden with the greatest occurrence among African American females (35–80/100,000)^2,3^. The disease commonly affects the respiratory and lymphatic systems, the skin, and the eyes; however, in 5% of patients the disease affects the nervous system, as a clinical entity defined as “neurosarcoidosis”^4^. This rare diagnosis has long been a challenge for clinicians, especially in the absence of systemic disease^5^.

The variable clinical manifestations of neurosarcoidosis depend on the size and localization of the granulomatous inflammation. Reportedly, symptoms range from effects on a single cranial nerve, mainly the optic nerve, to multiple cranial nerves or an involvement of the hypothalamic–pituitary axis, meninges, parenchyma of the brain and spinal cord, and occasionally the peripheral nervous system^6^. Laboratory investigations typically probe for elevated serum angiotensin converting enzyme (ACE), hypercalcemia, cerebrospinal fluid (CSF) abnormalities (e.g., elevated protein), pleocytosis, or increased ACE, IgG index, and T lymphocyte CD4^+^/CD8^+^ ratios. However, these are non-specific findings, and although they may support the diagnosis, the same findings may just as likely occur in other neuroinflammatory disorders^7,8^. Magnetic resonance imaging (MRI) studies of the central nervous system (CNS) often detect the presence of inflammation through diffuse or rim contrast enhancement (CE)^9^. Nevertheless, the findings are once again non-specific, and the granulomatous inflammation can be difficult to distinguish from other disease mimics. Tissue biopsy from the central or peripheral nervous system is the gold standard for diagnosis, but performing this can be challenging, given the invasiveness and significant morbidity associated with the procedure^10^.

Yet another contributing factor to the complexity of diagnosing suspected neurosarcoidosis is the variety of imitators, the most common of which are multiple sclerosis, neuromyelitis optica spectrum disorders, IgG4-related disease, systemic lupus erythematosus, Sjögren syndrome, and isolated angiitis of the CNS. Other well-known mimics are neoplastic (e.g., glioma, lymphoma, and meningioma) and infectious disorders. The latter emphasizes the importance of a comprehensive search for mycobacterial and fungal infections, as these are the most common causes of granuloma worldwide^11,12^.

Recognizing the complex clinicopathologic differential diagnosis of neurosarcoidosis and the difficulties concerning CNS biopsy, we report our findings and experiences in diagnosing neurosarcoidosis in a Scandinavian tertiary referring center and highlight the ongoing challenges, focusing on the diagnostic workup.

## Methods

This was a retrospective study of all adult patients registered at the Department of Neurology in Uppsala University Hospital in Sweden with a suspicion of neurosarcoidosis from 1990 to 2021. Most patients satisfied the diagnostic codes “Sarcoidosis unspecified, 135X” (ICD 9, 1990–1996) and “Sarcoidosis of other sites, D86.8” (ICD 10, 1997–2021). All patients fulfilled the diagnostic classification criteria (definite, probable, and possible neurosarcoidosis) first proposed by Zajicek et al. and later updated by Marangoni^5,13^ and the Neurosarcoidosis Consortium Consensus Group (Supplementary Table) ^6^. We identified 90 patients of northern European ancestry, 54 with possible neurosarcoidosis and 36 with probable neurosarcoidosis. However, CNS biopsy presented an alternative diagnosis in 24 patients initially classified as “possible neurosarcoidosis.” Therefore, the present study included only 66 patients.

Electrophysiological recording in terms of nerve conduction studies and electromyography (EMG) have long been standard examinations for disorders of the peripheral nervous system. Magnetic resonance imaging (MRI) of the CNS has been used systematically for patients with a suspicion of neurosarcoidosis since 1995. For radiological examinations, the statements from a neuroradiologist were dichotomized as normal/abnormal. Since 2007, several imaging approaches aimed at searching for extra-neural biopsy sites have been progressively introduced, including whole-body positron emission tomography/computed tomography (PET/CT) scanning with fluorodeoxyglucose (FDG) and thoracic high-resolution computed tomography (HRCT). Concomitantly, a comprehensive laboratory diagnostic workup was launched and progressively updated to exclude the main possible differential diagnoses of granuloma formation, with a focus on inflammatory, infectious, malignant, and systemic autoimmune diseases (Table 1).

**Table 1:** The comprehensive laboratory tests for patients with suspected neurosarcoidosis since 2007.

Blood sample studies	CSF studies	Viral and bacterial infections
Erythrocyte sedimentation rate (ESR)	Cytology	Borrelia (Lyme disease)
Complete blood cell counts	Flow cytometry	Rickettsia
Calcium, albumin	CD4/CD8	Bartonella
Serum creatinine	Cultures	Listeria
Sodium, Potassium	Leucocytes	Fungi: Cryptococcosis neoformans, histoplasmosis (stain and culture)
Liver function tests	Protein	Toxoplasma
IgG- subsets	Lactate	Mycobacteria (stain and culture)
P- Glucose	Glucose	Herpes simplex type 1 & 2, Cytomegalovirus (CMV), Epstein-Barr virus, Varicella Zoster virus, Enterovirus
Angiotensin Converting Enzyme (ACE)	Glial fibrillary acidic protein (GFAP)	Human Herpesvirus-6 (HHV-6)
IgG4, IgG-E	Neurofilament	Hepatitis
Thyroid-Stimulating Hormone (TSH), T4, T3, Anti-thyroid peroxidase antibodies		Human Immunodeficiency virus (HIV) syphilis
Vasculitis: ANA, ANCA, SSA, SSB	Electrophoresis	16srRNA, only in CSF
Rheumatoid factor, Cardiolipin antibodies, B2-glycoprotein-1	ACE	*In selected cases*
Lupus anticoagulant	Neuronal antibodies	*Human T-lymphotropic virus type I and II (HTLV-1, II)*
Cryoglobulins, complement screening		*Acanthamoeba, cysticercosis, leprosy, helminthic infection*
Aquaporin-4		*Opportunistic infections in immunosuppressed patients (JC virus)*
Myelin oligodendrocyte glycoprotein (MOG)-antibodies		
Hormone evaluation in case of pituitary and hypothalamic involvement		
Neuronal antibodies		

Because different laboratories and methods were used throughout the duration of the study, a given test was interpreted according to the reference ranges established at the time it was performed. The CSF examination, in terms of pleocytosis, elevated protein (≥ 0.5 g/l), lactate, glucose, and IgG-index remained relatively similar, but the reference level for increased angiotensin converting enzyme (ACE) in the CSF has changed over the years. A CSF T lymphocyte CD4^+^/CD8^+^ ratio ratios ≥ 5 was considered a cut-off level for suggesting neurosarcoidosis. The reference level for increased serum ACE, lysozyme (an enzyme found in neutrophilic granulocytes and monocytes), and ionized calcium remained unchanged throughout the study period. We did not employ serum or CSF sIL-2R because it lacks specificity. A study has shown elevated CSF-Soluble interleukin-2R in > 50% of patients with neurosarcoidosis; however, IL-2R may also be elevated in CNS lymphoma and neurotuberculosis^14^. Pathologically, a granuloma is defined by a highly differentiated population of mononuclear macrophages with CD4^+^ T lymphocytes in the center and CD8^+^ T lymphocytes in the peripheral zone^15,16^.

The medical records for each patient were individually reviewed to obtain demographic characteristics, clinical manifestations, findings from MRI studies of the brain and spinal cord, thoracic HRCT, FDG- PET/CT, nerve conduction studies, electromyography studies, laboratory investigations including CSF and serum analyses, CNS and extra-neural tissue biopsy reports, as well as data on systemic involvement and comorbidity.

### Statistical analysis

Continuous variables were recorded as medians (range/IQR). Dichotomous variables were examined by frequency distribution and recorded as proportions. The statistical analysis was performed using IBM SPSS (version 28).

### Ethical consent

The study was conducted in accordance with the guidelines set by the Declaration of Helsinki and was approved by the Ethical Review committee in Uppsala, Sweden (Regionala Etikprövningsnämnden i Uppsala,) (reference number: Dnr 2018/429, Dnr 2021-06684-02). Due to the retrospective design of the study, patient informed consent was waived by the Ethical Review committee in Uppsala, Sweden (Regionala Etikprövningsnämnden i Uppsala).

## Results

### Result 1. The most common clinical feature was cranial neuropathy

The median age at the onset of symptoms was 49 years. The patient cohort had a similar proportion of males and females, and 25% were smokers. Interestingly, almost 50% of the patients presented as a medical emergency. The most frequently occurring clinical signs were cranial neuropathies (n = 25), in which the optic nerve was mostly affected (n = 15). Motor deficits (n = 21), hydrocephalus (n = 7), and seizures (n = 7) were among other presenting symptoms (Table 2). Features of pituitary/hypothalamic dysfunction, included diabetes insipidus, hypothyroidism, ACTH and prolactin deficiency, and visual field loss (bitemporal hemianopsia). Headache (n = 11) was frequently seen both in patients with leptomeningeal CE and pituitary dysfunction. Vertigo (n = 10), nausea (n = 4), chronic fever (n = 4), cognitive decline, confusion, sexual dysfunction, and hallucinations (n = 2 each) were also encountered. The peripheral nervous system was affected in three patients, who were documented with small-fiber neuropathy and two with axonal sensory motor polyneuropathy.

**Table 2:** Demographic and clinical features of patients with neurosarcoidosis (n = 66).

Patients, n (%)	66 (100)
Gender, male/female	31/35 (47/53)
Caucasian (northern ancestry)	66 (100)
Age at symptom onset, median (IQR), years	49 (35–60)
Systemic sarcoidosis	
Pulmonary/other locations	29/7 (43/11)
Active smoking	17 (25)
Source of referral	
Emergency Department	30 (45)
Neurosurgery Clinic	13 (20)
General practitioner	13 (20)
Other clinics (Endocrinology/Ophthalmology/Rheumatology)	10 (15)
Symptom onset	
Mild	34 (51)
Sudden and severe	32 (49)
Clinical manifestations	
Cranial nerves II, III, VII, VIII, VIX, X, XII	25 (38)
Motor deficit (hemi/paraparesis)	21 (32)
Optic neuropathy, papilledema	15 (22)
Pituitary/hypothalamic dysfunction	8 (12)
Hydrocephalus	7 (10)
Iritis, Uveitis	5 (7.5)
Polyneuropathy	5 (7.5)
Blindness	5 (7.5)
Ataxia	5 (7.5)
Limb sensory disturbance	5 (7)
Hearing impairment	3 (4.5)
Dysphagia	3 (4.5)
Nystagmus	2 (3)
Aphasia	1 (1.5)

Thirty-six patients (55%) had extra neurological target organ involvement, with the vast majority (29/36) presenting with pulmonary sarcoidosis. A wide spectrum of extra-pulmonary and extra-CNS disease was found, including cutaneous and pulmonary (n = 2); cutaneous, parotid and pulmonary (n = 1); cutaneous parotid and peripheral lymph node (n = 1); cutaneous, epiglottis, heart, joint and pulmonary (n = 1); bone marrow, peripheral lymph node, and pulmonary (n = 1); and gastric (n = 1).

### Result 2. Leptomeningeal contrast enhancement and isolated pituitary-hypothalamic axis involvement were the most striking findings on MRI

**Figure 1:**
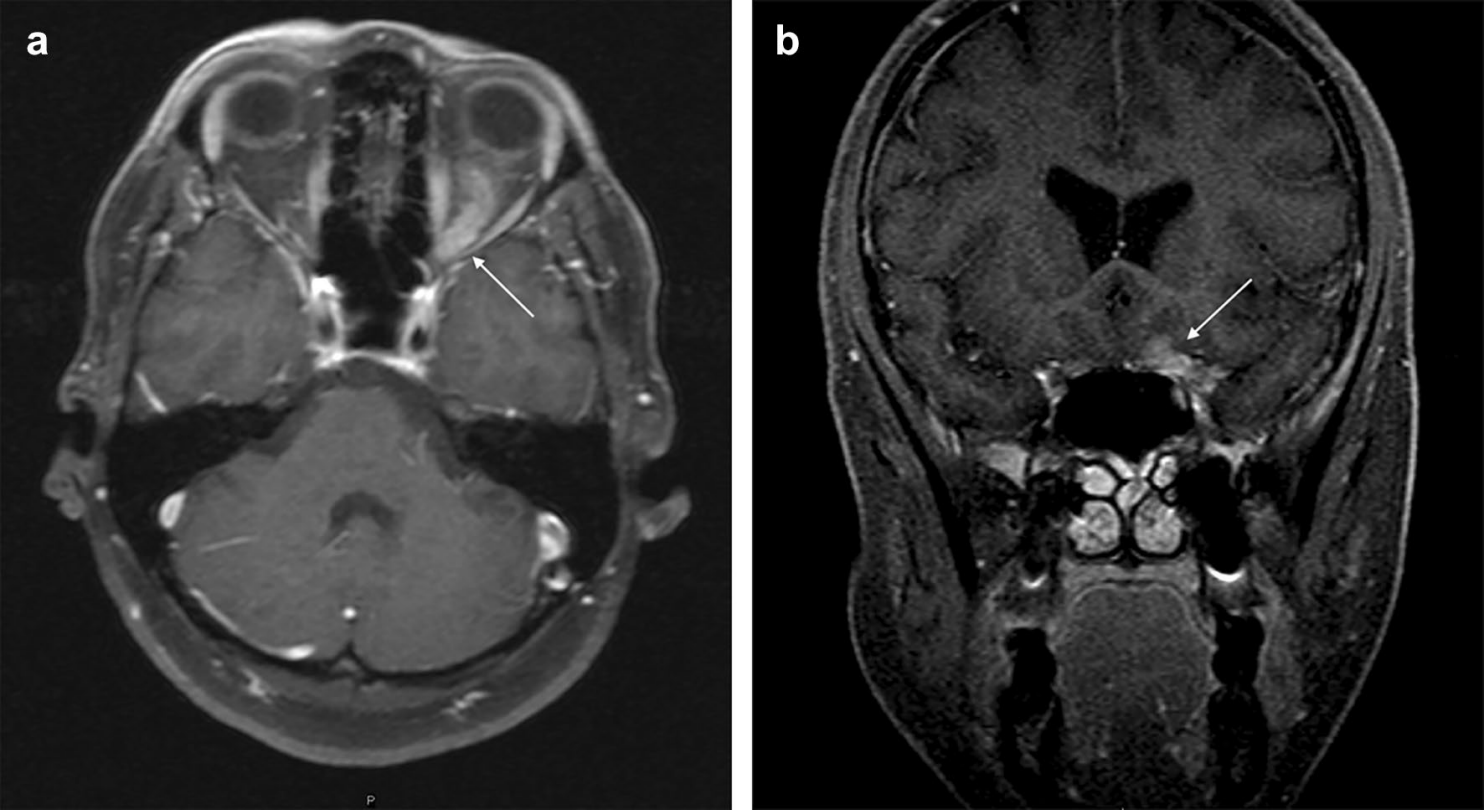
Sarcoidosis engaging optic nerves. (**a**) Post-contrast T1-weighted axial image shows diffuse enhancement and thickening of the left intra orbital optic nerve sheath in a 48-year-old female presenting with progressive vision loss first in left eye concerning for optic glioma. Upon biopsy a diagnosis of definite optic sarcoidosis was made. A few months later, she presented with progressive vision loss in the right eye. Receiving immunotherapy, the vision has been stable for 13 years. (**b**) Cor T1 fs Gd Shows intracranial extension of contrast enhancement around the left optic nerve.

**Figure 2:**
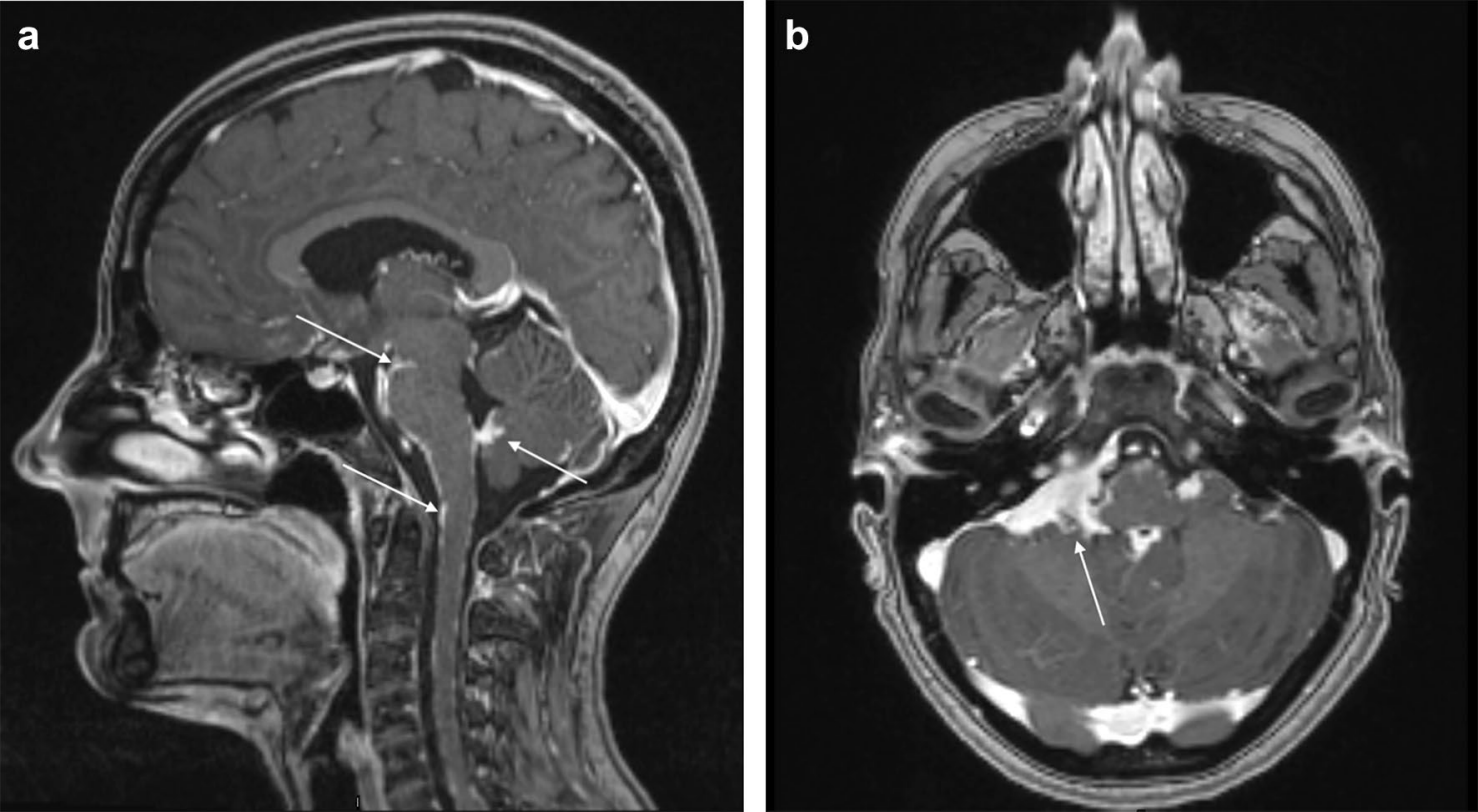
Sarcoidosis in the base of the brain. (**a**) Sagittal T1Gd shows multifocal and diffuse linear leptomeningeal contrast enhancement predominant in the basal cisterns and in the posterior fossa and even along the cervical spinal cord in a 29-year-old woman with definite neurosarcoidosis. (**b**) Axial T1 Gd shows a contrast enhancing extra-axial mass in the right cerebellopontine angle.

**Figure 3:**
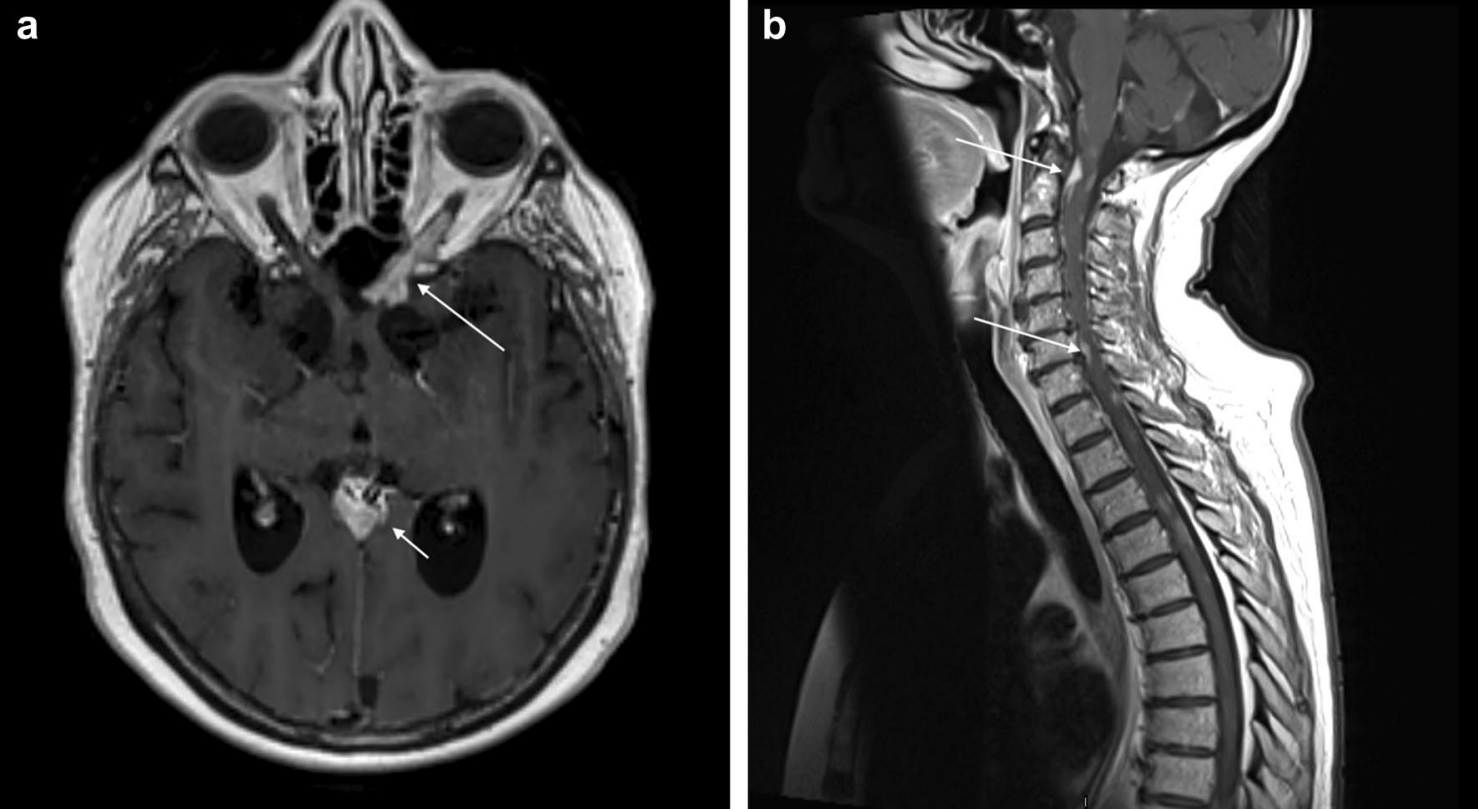
Spinal sarcoidosis. (**a**) Axial T1Gd shows diffuse enhancement along posterior intra orbital segment of left optic nerve with intracranial extension and nodular enhancement along the retroorbital optic nerve. In addition, nodular enhancement around pineal gland and the vein of Galen. (**b**) Sagittal T1 Gd shows multiple contrast enhancing nodular end linear lesions along the brain stem and spinal cord in a 52 -year -old woman with definite neurosarcoidosis.

At the time of the first clinical observation, brain and spinal cord MRI were performed in 61 (94%) and 47 (52%) of patients, respectively. MRI imaging was unavailable in five patients from the late 1980s who presented with polyneuropathy. Leptomeningeal and dural gadolinium enhancement, intraparenchymal lesions (either supratentorial (35%) or infratentorial masses (15%)), and isolated pituitary gland and hypothalamic involvement (17%) were among the most common features. In the presence of CE, these features signal biologically active inflammation. More specifically, CE lesions most often affected the optic nerve. Figure 1a,b illustrate optic nerve enhancement, which was seen in 15 patients (22%). Examples of the involvement of basal cisterns, posterior fossa, cerebellopontine angle, pineal gland, and the vein of Galen are shown in Figs. 2a,b, and 3a, respectively. MRI of the spinal cord revealed leptomeningeal CE, intramedullary tumor-like lesions, and enlargement of the spinal cord due to edema (Fig. 3b).

FDG-PET/CT of the brain and whole body was performed in 22 (33%) patients. In 11 cases, we found an increased uptake in the mediastinal and other peripheral lymphatic nodes, apart from an increased uptake in enhanced lesions in the CNS (Table 3).

**Table 3:** Diagnostic work-up of patients with neurosarcoidosis (n = 66).

Examinations	No of procedures performed, n (%)	Abnormal results, n (%)
Brain MRI	61 (94)	61 (94)
Spinal MRI	47 (52)	15 (23)
Lumbar puncture	54 (82)	42 (77)
Missing	12 (18)	
CSF-White cell count, cells/L	54 (81)	31 (57)
Median, (IQR)	8.0 (0–35.2)	
Missing	9 (13)	
CSF-CD4/CD8 ratio, n (%) *cut-off* > *5*	18 (27)	9 (13)
Range	2.10–17.6	
Median (IQR)	5.5 (3.95–10)	
Missing	36 (54)	
CSF-ACE, n (%)	35 (53)	19 (28)
Range, U/L	0.10–12.0	
Median (IQR)	2.20 (2.0- 3.3)	
Missing	19 (28)	
CSF-oligoclonal bands, n (%)	41(62)	22 (40)
Missing	11 (16)	
CSF-protein, n (%)	42 (77)	24 (44)
Median (IQR), g/L	0.48 (0.30- 0.75)	
Missing	10 (15)	
Serum ACE *Reference range, (20–70 U/L)*	42 (63)	4 (6)
Serum calcium ionized *Reference range (1.051.30 mmol/L)*	38 (57)	4 (6)
Serum lysozyme *Reference range (9.6–16.8 U/mL)*	35 (53)	3 (4,5)
Bronchoalveolar lavage (BAL)	10 (15)	8 (12)
Thoracic high-resolution CT (HRCT)	50 (75)	45 (68)
FDG-PET (whole body)	22 (33)	11 (17)
Neurography, EMG	10 (15)	5 (7.5)
EEG	11 (16)	4 (6)

### Result 3. The CSF studies revealed unspecific intrathecal inflammation in 77% of the patients

A lumbar puncture was performed in 80% of our patients. Notably, in the early decades of this study, the first CSF findings were normal, mostly due to prior administration of corticosteroids. However, upon repetition after a steroid-free interval, the CSF findings usually became abnormal in these patients. Overall, 77% of the patients had abnormal CSF findings. The most striking features were raised leucocyte count (≥ 5 cells/mm^3^), lymphocytosis, and elevated CSF-T lymphocyte CD4^+^/CD8^+^ ratios, CSF-ACE, and CSF-oligoclonal bands (OCBs). All these findings suggest a neuroinflammatory disease. The presence of CSF-OCBs was usually associated with a raised WCC and an elevated CSF protein. The CSF/plasma glucose ratio was normal, except in one case. A lumbar puncture was not a part of the investigation in 12 patients, who were diagnosed with polyneuropathy (n = 5); severe visual impairment (n = 2), spinal tumor (n = 1). In the remaining four cases, the data were missing (Table 3).

Serum calcium and serum ACE were elevated in four patients, and serum lysozyme in three patients. Most importantly, the full diagnostic workup shown in Table 1 allowed for the exclusion of infection and other causes of neuroinflammation.

### Result 4. A CNS biopsy allowed for a correct diagnostic attribution in almost one-half of incorrectly classified “possible neurosarcoidosis” patients

The initial search identified 90 patients, introduced chronologically every five years from 1990 to 2021, illustrating an increased frequency of CNS biopsies in the last decade compared with the early period of the study (Table 4). Among 38 patients who underwent CNS biopsy, only four patients originated from the “probable” group; the remainder (n = 34) had been placed in the “possible” categories at initial assessment, based on their clinical presentations and the absence of systemic sarcoidosis. Among the patients classified as “possible neurosarcoidosis”, CNS biopsy revealed 14 patients (34%) with “definite neurosarcoidosis” and 24 patients (63%) with an alternative diagnosis. Demographic, clinical, and CNS biopsy data of the definite neurosarcoidosis patients (n = 14) revealed that the majority presented with isolated CNS lesions (Table 5). Notably, a CNS biopsy was waived in the majority of patients (n = 36) who were classified as "probable neurosarcoidosis" because of the presence of systemic sarcoidosis. In patients without systemic sarcoidosis, the cortical and isolated pituitary gland/dorsum sellae were among the biopsy sites that most frequently yielded non-neurosarcoidosis conditions, allowing for a precise diagnosis. In total, 10 of the 16 patients who initially presented with isolated pituitary lesions were biopsied. Eight patients were excluded from the cohort because of diagnoses other than neurosarcoidosis. Ultimately, sixty-six patients (35 females and 31 males) fulfilled the diagnostic criteria for definite (n = 14), probable (n = 32), and possible (n = 20) neurosarcoidosis (Table 4).

**Table 4:** Diagnostic classification of neurosarcoidosis according to date of diagnosis and CNS biopsy.

Date of diagnosis	Classification according to Consensus diagnostic criteria					
	Initial diagnosis		Final diagnosis			
	–		No CNS biopsy		CNS biopsy	
	Possible NS	Probable NS	Possible NS	Probable NS	Definite NS (pre-biopsy classification)	Other diagnoses (pre-biopsy classification)
1990–1994	1	3	0	3	1 (1 possible)	–
1995–1999	4	3	2	3	2 (2 possible)	–
2000–2004	3	4	2	4	–	1 not conclusive (possible)
2005–2009	7	3	3	1	4 (2 probable) (2 possible)	1 CNS lymphoma 1 not conclusive (all possible)
2010–2014	12	13	5	12	1 (1 probable)	2 CNS lymphoma 4 IgG4-related disease 1 Granular tumor (All possible)
2015–2019	24	9	8	8	5(1 probable) (4 possible)	2 CNS lymphoma 2 IgG4-related disease 3 not conclusive 1 metastasis 1 granular tumor 1 xanthomatous hypophysitis 1 abscess 1 Wegener Granulomatosis (All possible)
2020–2021	3	1	0	1	1 (1 possible)	1 not conclusive 1 Astrocytoma (all possible)
Total	54	36	20	32	14 (10 possible) (4 probable)	24 (all possible)

**Table 5:** Demographic, clinical, and CNS biopsy findings in patients with definitive neurosarcoidosis (n = 14).

Patient No./Age at onset (years) Sex	Initial diagnosis	Presenting symptom	Systemic involvement Co-morbidity	CSF findings	Deep medullary sign (MRI)	Biopsy site	Final histopathology diagnosis
1/45 Female	Possible NS	Hydrocephalus, blindness	Anti-phospholipid syndrome Diabetes Hypothyroid	Pleocytosis Elevated protein Oligoclonal bands (OCBs)	Absent	Optic nerve	Non-caseating granuloma
2/21 Female	Possible NS	Hydrocephalus	–	Elevated protein OCBs	Absent	Meninges The foramen of Monro	Non-caseating granuloma
3/60 Female	Probable NS	Dizziness, deafness, Epilepsy	Myocardial sarcoidosis, Systemic lupus Erythematous	Unremarkable	Absent	Internal auditory canal	Non-caseating granuloma
4/50 Female	Probable NS	Cognitive impairment, epilepsy	Pulmonary sarcoidosis	Pleocytosis Elevated protein OCBs	Absent	Temporal lobe	Non-caseating granuloma
5/48 Female	Possible NS	Unilateral blindness	–	Data not available	Absent	Optic nerve	Non-caseating granuloma
6/31 Female	Possible NS	Dysphagia, dysphonia, diplopia	–	Pleocytosis Elevated protein OCBs	Absent	Meninges	Non-caseating granuloma
7/57 Female	Possible NS	Pituitary dysfunction Visual field loss	IgG4 syndrome	Pleocytosis Elevated protein	Absent	Meninges, pituitary gland	Non-caseating granuloma
8/63 Male	Possible NS	Hemiparesis	Diabetes	Pleocytosis Elevated protein OCBs	Absent	Internal capsule	Non-caseating granuloma
9/60 Female	Possible NS	Epilepsy, Multiple cranial nerve affection	Pulmonary sarcoidosis, Diabetes	Data not available	Absent	Meninges	Non-caseating granuloma
10/52 Male	Possible NS	Paraparesis	–	Pleocytosis Elevated protein OCBs	Absent	Spinal cord	Non-caseating granuloma
11/19 Female	Possible NS	Pituitary dysfunction	No	Unremarkable	Absent	Hypo thalamus	Granuloma
12/69 Female	Probable NS	Hydrocephalus,Uveitis	Pulmonary and ocular sarcoidosis	Pleocytosis Elevated protein Elevated ACE	Absent	Meninges	Non-caseating granuloma
13/64 Female	Probable MS	Myelopathy, unilateral blindness	Skin, lung, and epiglottis sarcoidosis	Pleocytosis Elevated protein Elevated ACE OCBs	Absent	Temporal lobe	Non caseating granuloma
14/45 Male	Possible NS	Nausea, dizziness, Paraparesis	–	Elevated lactate	Absent	Spinal Meninges	Non-caseating granuloma

## Discussion

In 2018, the Neurosarcoidosis Consortium Consensus Group proposed a table of possible differential diagnoses and their investigations, depending on the clinical context^6^. The diagnostic workup for the patients included in this study has largely followed this approach since 2007.

The most common symptom in our cohort was cranial neuropathy, mainly presenting as optic neuropathy, hydrocephalus, and motor signs. These findings are consistent with previously reported clinical manifestations of neurosarcoidosis^17,18^. Strikingly, 17% of the patients presented with isolated pituitary/hypothalamic dysfunction. A recent study reported an involvement of the hypothalamus/pituitary axis in 9% of patients with suspected neurosarcoidosis^19^.

Hypothalamus, pituitary gland, and optic chiasma are among the predilection sites for neurosarcoidosis. Patients belonging to this category usually present with features of hypopituitarism such as central diabetes insipidus, hyperprolactinemia, and other endocrinopathies arising from inflammation of the pituitary gland or a sellar mass lesion^20,21^. Gadolinium enhanced MRI of the pituitary gland shows either asymmetrical pituitary enlargement with stalk deviation, as in the case of adenoma, or symmetrical enlargement of the pituitary gland and pituitary stalk, as in the case of lymphocytic hypophysitis. Another scenario is a normal MRI of the pituitary gland which makes the diagnosis of neurosarcoidosis even more challenging. As was the case for our patients, the histopathology of hypophysitis was due to sarcoidosis (n = 2), IgG4-related disease (n = 5), xanthogranulomatous (n = 1), or lymphocytic hypophysitis (n = 2) affecting different parts of or the entire pituitary gland^22^.

All our patients with large- and small-fiber neuropathy (7%) had systemic disease, raising the suspicion of peripheral nervous system involvement. This is in line with the decision of the 2018 Neurosarcoidosis Consortium Consensus Group, which ultimately included the peripheral nervous system as a feature of neurosarcoidosis^6^.

Given the rarity of isolated neurosarcoidosis, an open-minded discussion of the differential diagnosis of each MRI pattern is crucial in every single case, as is correlating the findings to patient history and other laboratory findings. The lack of reliable biomarkers compelled us to rely heavily on repeated MRI studies (normally twice per year) in combination with clinical findings, both at the onset and later on, for monitoring disease activity. The MRI studies in this cohort revealed a wide range of findings, including leptomeningeal, dural, and intraparenchymal lesions with CE, as well as involvement of the pituitary gland, hypothalamus, cranial nerves, and hydrocephalus. All these findings were in agreement with previous reports^19,23^. Spinal involvement was observed in 23% of our sample, mostly as leptomeningeal enhancement^24,25^. Notably, the deep medullary vein sign on MRI was absent in all our patients with histological features of neurosarcoidosis (n = 14), despite a reported sensitivity of 71.4% and a specificity of 92.3% for the diagnosis of neurosarcoidosis^26,27^.

A careful assessment of the systemic manifestations is an important part of the diagnostic work-up. This justifies the use of whole body ^18^F-FDG PET/CT for visualization of extra- neural sites of inflammatory active sarcoidosis suitable for diagnostic biopsy, for detection of clinically silent lesions such as ocular or cardiac sarcoidosis, and for evaluation of the treatment response^28,29^. In our study, FDG PET/CT was part of the investigation in 33% of the cases. The results were used to identify and obtain biopsies from sites with hypermetabolism.

The most striking laboratory findings in our cohort was elevated levels of WCC, protein, T lymphocyte CD4^+^/CD8^+^ ratios, ACE, and OCBs in the CSF, suggesting a neuroinflammatory disease. The frequency of OCBs in our cohort was consistent with previous reports (27–37%)^5^. Recent data showed that a combined CD4^+^/CD8^+^ ratio ≥ 5 and elevated WCC had a negative predictive value of 88%, with a specificity of 95% for neurosarcoidosis^30,31^. By contrast, serum ACE measurements lack both sensitivity and specificity, as ACE levels can also be elevated in other diseases, such as diabetes mellitus, hypothyroidism, and lymphoma^32^. Overall, serum biomarkers have only limited value in the diagnostic work-up of neurosarcoidosis. The ACE levels in the CSF, although more specific (94–95%) than in the serum, are rather insensitive (24–55%). The level increases in proportion to that of CSF protein and is reported to increase in CNS infections and malignant tumors^33^.

One study showed elevated levels of lysozyme and B2-microglobulin in the CSF in a small sample of patients with neurosarcoidosis, but those findings were not confirmed later, and the diagnostic value of both serum and CSF lysozyme levels remains doubtful^34^. Given that 23% of our patients had normal CSF and that no specific pattern is diagnostic, our CSF analyses were conducted mainly to confirm neuroinflammation.

Because the other tests were never conclusive enough to make a definitive diagnosis of neurosarcoidosis, performing a greater number of CNS biopsies will increase the chance of achieving a definite diagnosis. However, CNS biopsies were not the first option unless patients were first given a standardized noninvasive investigation (e.g., blood, CSF, MRI, PET imaging) and/or if a very aggressive or atypical course was present. Examples included hydrocephalus, rapid deterioration of neurological functions, mass lesions, multiple cranial nerve palsies, and pituitary enlargement, which signaled a clear indication for CNS biopsy. Initiating treatment with high-dose steroids for a subset of patients with severe neurological symptoms before all investigations had been completed led to a diagnosis of non-specific chronic inflammation on biopsy, thereby complicating the histopathological confirmation of neurosarcoidosis.

The diagnosis of "probable neurosarcoidosis" is the most common scenario in clinical practice, as soon as the investigation reveals signs of systemic sarcoidosis and excludes other possible diagnoses. Only four cases belonging to this category were biopsied, compared to the “possible neurosarcoidosis” group, most likely because the diagnostic certainty was considered higher and the CNS manifestations were attributed to systemic sarcoidosis. A high suspicion of neoplasm in solitary intracranial or intraspinal mass lesions, a threatening hydrocephalus in need of a ventriculoperitoneal shunt, or the presence of cortical tumor-like lesions, especially in the temporal lobe, causing medically refractory epilepsy, were among the clinically precarious situations that justified the need for CNS biopsy despite histologically confirmed active systemic sarcoidosis.

In reality, the most difficult scenario was investigating isolated neurosarcoidosis. A comprehensive diagnostic workup had two purposes: first, to reduce the rate of incorrectly diagnosing patients with “possible neurosarcoidosis”, and second, to identify other CNS disease mimics that needed urgent treatment.

We recognize a number of limitations of this study. One is its retrospective nature; another is the small number of patients, which is explained by the low overall prevalence of neurosarcoidosis. A risk of selection bias also probably existed because the more severe cases were referred to our center. Furthermore, this study spanned the years between 1990 and 2021, and diagnostic tools have vastly improved over that period.

## Conclusions

The high number of patients with probable (49%) and possible (30%) diagnosis versus only 21% of cases with definite neurosarcoidosis reflect real world data. Neurosarcoidosis is a rare heterogeneous disorder, and because of the morbidity associated with CNS biopsy, the diagnosis relies on how well the clinician interprets the likelihood of differential diagnosis based on the patient´s history, clinical findings, results of neuroimaging, and CSF and serum analyses.

## Supplementary Information

Supplementary Table S1.

## Data Availability

The datasets used and/or analyzed during the current study available from the corresponding author on reasonable request.
