# Supplementary material for: A comprehensive diagnostic approach in suspected neurosarcoidosis
Source: Sci Rep. 2023 Apr 21;13:6539. doi: 10.1038/s41598-023-33631-z (PMC10121682; doi:10.1038/s41598-023-33631-z)
Supplement: Supplementary file 1 — Supplementary Table S1. [file 41598_2023_33631_MOESM1_ESM.docx]

**Supplementary Table (S1)**

Recommended diagnostic criteria according to The Neurosarcoidosis Consortium Consensus Group

| Possible | Clinical findings and MRI, CSF, and EMG findings are compatible with granulomatous inflammation of the nervous system and other causes are excluded by detailed investigations. - There is no pathologic confirmation of granulomatous disease |
| --- | --- |
| Probable | Clinical findings and MRI, CSF, and EMG findings are compatible with granulomatous inflammation of the nervous system and other causes are excluded by detailed investigations. - There is confirmation of systemic granulomatous disease consistent with sarcoidosis |
| Definite | Clinical findings and MRI, CSF and EMG findings are compatible with granulomatous inflammation of the nervous system and other causes are excluded by detailed investigations. - Nervous system pathology is consistent with neurosarcoidosis.  Type a: Extraneural sarcoidosis.  Type b: No extraneural sarcoidosis (isolated CNS sarcoidosis). |
